# Associations of Educational Attainment, Occupation, Social Class and Major Depressive Disorder among Han Chinese Women

**DOI:** 10.1371/journal.pone.0086674

**Published:** 2014-01-31

**Authors:** Jianguo Shi, Yan Zhang, Feihu Liu, Yajuan Li, Junhui Wang, Jonathan Flint, Jingfang Gao, Youhui Li, Ming Tao, Kerang Zhang, Xumei Wang, Chengge Gao, Lijun Yang, Kan Li, Shenxun Shi, Gang Wang, Lanfen Liu, Jinbei Zhang, Bo Du, Guoqing Jiang, Jianhua Shen, Zhen Zhang, Wei Liang, Jing Sun, Jian Hu, Tiebang Liu, Xueyi Wang, Guodong Miao, Huaqing Meng, Yi Li, Chunmei Hu, Yi Li, Guoping Huang, Gongying Li, Baowei Ha, Hong Deng, Qiyi Mei, Hui Zhong, Shugui Gao, Hong Sang, Yutang Zhang, Xiang Fang, Fengyu Yu, Donglin Yang, Tieqiao Liu, Yunchun Chen, Xiaohong Hong, Wenyuan Wu, Guibing Chen, Min Cai, Yan Song, Jiyang Pan, Jicheng Dong, Runde Pan, Wei Zhang, Zhenming Shen, Zhengrong Liu, Danhua Gu, Xiaoping Wang, Xiaojuan Liu, Qiwen Zhang, Yihan Li, Yiping Chen, Kenneth S. Kendler

**Affiliations:** 1 Xian Mental Health Center, New Qujiang District, Xian, Shaanxi, People’s Republic of China; 2 The Affiliated Mental Health Institute of Xi’an School of Medicine, Xian, Shaanxi, People’s Republic of China; 3 Wellcome Trust Centre for Human Genetics, Oxford, United Kindgom; 4 Chinese Traditional Hospital of Zhejiang, Hangzhou, Zhejiang, People’s Republic of China; 5 No.1 Hospital of Zhengzhou University, Zhengzhou, Henan, People’s Republic of China; 6 Xinhua Hospital of Zhejiang Province, Hangzhou, Zhejiang, People’s Republic of China; 7 No.1 Hospital of Shanxi Medical University, Taiyuan, Shanxi, People’s Republic of China; 8 ShengJing Hospital of China Medical University, Heping District, Shenyang, Liaoning, People’s Republic of China; 9 No. 1 Hospital of Medical College of Xian Jiaotong University, Xian, Shaanxi, People’s Republic of China; 10 Jilin Brain Hospital, Siping, Jilin, People’s Republic of China; 11 Mental Hospital of Jiangxi Province, Nanchang, Jiangxi, People’s Republic of China; 12 Shanghai Mental Health Center, Shanghai, People’s Republic of China; 13 Beijing Anding Hospital of Capital University of Medical Sciences, Deshengmen wai, Xicheng District, Beijing, People’s Republic of China; 14 Shandong Mental Health Center, Jinan, Shandong, People’s Republic of China; 15 No. 3 Hospital of Sun Yat-sen University, Tianhe District, Guangzhou, Guangdong, People’s Republic of China; 16 Hebei Mental Health Center, Baoding, Hebei, People’s Republic of China; 17 Chongqing Mental Health Center, Jiangbei District, Chongqing, People’s Republic of China; 18 Tianjin Anding Hospital, Hexi District, Tianjin, People’s Republic of China; 19 No.4 Hospital of Jiangsu University, Zhenjiang, Jiangsu, People’s Republic of China; 20 Psychiatric Hospital of Henan Province, Xinxiang, Henan, People’s Republic of China; 21 Nanjing Brain Hospital, Nanjing, Jiangsu, People’s Republic of China; 22 Harbin Medical University, Nangang District, Haerbin, Heilongjiang, People’s Republic of China; 23 Shenzhen Kang Ning Hospital, Luohu District, Shenzhen, Guangdong, People’s Republic of China; 24 First Hospital of Hebei Medical University, Shijiazhuang, Hebei, People’s Republic of China; 25 Guangzhou Brain Hospital (Guangzhou Psychiatric Hospital), Liwan District, Guangzhou, Guangdong, People’s Republic of China; 26 No.1 Hospital of Chongqing Medical University, Yuzhong District, Chongqing, People’s Republic of China; 27 Dalian No.7 Hospital, Ganjingzi District, Dalian, Liaoning, People’s Republic of China; 28 No.3 Hospital of Heilongjiang Province, Beian, Heilongjiang, People’s Republic of China; 29 Wuhan Mental Health Center, Wuhan, Hubei, People’s Republic of China; 30 Sichuan Mental Health Center, Mianyang, Sichuan, People’s Republic of China; 31 Mental Health Institute of Jining Medical College, Dai Zhuang, Bei Jiao, Jining, Shandong, People’s Republic of China; 32 Liaocheng No.4 Hospital, Liaocheng, Shandong, People’s Republic of China; 33 Mental Health Center of West China Hospital of Sichuan University, Wuhou District, Chengdu, Sichuan, People’s Republic of China; 34 Suzhou Guangji Hospital, Suzhou, Jiangsu, People’s Republic of China; 35 Anhui Mental Health Center, Hefei, Anhui, People’s Republic of China; 36 Ningbo Kang Ning Hospital, Zhenhai District, Ningbo, Zhejiang, People’s Republic of China; 37 Changchun Mental Hospital, Changchun, Jilin, People’s Republic of China; 38 No.2 Hospital of Lanzhou University, Lanzhou, Gansu, People’s Republic of China; 39 Fuzhou Psychiatric Hospital, Cangshan District, Fuzhou, Fujian, People’s Republic of China; 40 Harbin No.1 Special Hospital, Haerbin, Heilongjiang, People’s Republic of China; 41 Jining Psychiatric Hospital, North Dai Zhuang,Rencheng District, Jining, Shandong, People’s Republic of China; 42 No.2 Xiangya Hospital of Zhongnan University, Furong District, Changsha, Hunan, People’s Republic of China; 43 Xijing Hospital of No.4 Military Medical University, Xian, Shaanxi, People’s Republic of China; 44 Mental Health Center of Shantou University, Shantou, Guangdong, People’s Republic of China; 45 Tongji University Hospital, Shanghai, People’s Republic of China; 46 Huaian No.3 Hospital, Huaian, Jiangsu, People’s Republic of China; 47 Huzhou No.3 Hospital, Huzhou, Zhejiang, People’s Republic of China; 48 Mudanjiang Psychiatric Hospital of Heilongjiang Province, Xinglong, Mudanjiang, Heilongjiang, People’s Republic of China; 49 No.1 Hospital of Jinan University, Guangzhou, Guangdong, People’s Republic of China; 50 Qingdao Mental Health Center, Shibei District, Qingdao, Shandong, People’s Republic of China; 51 Guangxi Longquanshan Hospital, Yufeng District, Liuzhou, People’s Republic of China; 52 Daqing No.3 Hospital of Heilongjiang Province, Ranghulu district, Daqing, Heilongjiang, People’s Republic of China; 53 Tangshan No.5 Hospital, Lunan District, Tangshan, Hebei, People’s Republic of China; 54 Anshan Psychiatric Rehabilitation Hospital, Lishan District, Anshan, Liaoning, People’s Republic of China; 55 Weihai Mental Health Center, ETDZ, Weihai, Shandong, People’s Republic of China; 56 Renmin Hospital of Wuhan University, Wuchang District, Wuhan, Hubei, People’s Republic of China; 57 Tianjin First Center Hospital, Hedong District, Tianjin, People’s Republic of China; 58 Hainan Anning Hospital, Haikou, Hainan, People’s Republic of China; 59 Clinical Trial Service Unit, Richard Doll Building, Oxford, United Kingdom; 60 Virginia Institute for Psychiatric and Behavioral Genetics, Department of Psychiatry, Virginia Commonwealth University, Richmond, Virginia, United States of America; University of Iowa Hospitals & Clinics, United States of America

## Abstract

**Background:**

The prevalence of major depressive disorder (MDD) is higher in those with low levels of educational attainment, the unemployed and those with low social status. However the extent to which these factors cause MDD is unclear. Most of the available data comes from studies in developed countries, and these findings may not extrapolate to developing countries. Examining the relationship between MDD and socio economic status in China is likely to add to the debate because of the radical economic and social changes occurring in China over the last 30 years.

**Principal findings:**

We report results from 3,639 Chinese women with recurrent MDD and 3,800 controls. Highly significant odds ratios (ORs) were observed between MDD and full time employment (OR = 0.36, 95% CI = 0.25–0.46, logP = 78), social status (OR = 0.83, 95% CI = 0.77–0.87, logP = 13.3) and education attainment (OR = 0.90, 95% CI = 0.86–0.90, logP = 6.8). We found a monotonic relationship between increasing age and increasing levels of educational attainment. Those with only primary school education have significantly more episodes of MDD (mean 6.5, P-value = 0.009) and have a clinically more severe disorder, while those with higher educational attainment are likely to manifest more comorbid anxiety disorders.

**Conclusions:**

In China lower socioeconomic position is associated with increased rates of MDD, as it is elsewhere in the world. Significantly more episodes of MDD occur among those with lower educational attainment (rather than longer episodes of disease), consistent with the hypothesis that the lower socioeconomic position increases the likelihood of developing MDD. The phenomenology of MDD varies according to the degree of educational attainment: higher educational attainment not only appears to protect against MDD but alters its presentation, to a more anxious phenotype.

## Introduction

One of the more robust findings in the literature describing the risk factors for psychiatric disorders concerns the association between lower socio-economic position (SEP) and higher prevalence rates of illness [1], though the nature of the relationship is believed to differ among disorders [2]. In particular, the role that SEP plays in major depressive disorder (MDD), psychiatry’s commonest disorder and the world’s third leading contributor to the global burden of disease [3], has been a matter of both interest and contention. For instance, in some studies the association between educational status and MDD is not stable [4], [5].

Cross-sectional studies show a consistent relationship between low SEP and increased prevalence of MDD but the nature of that relationship is unclear. Higher rates of MDD among those in lower SEP might be observed because episode duration is longer or because of higher incidence rates; indeed, some have argued that if low SEP causes MDD, then we expect those in low SEP categories to experience more episodes [6]. A meta-analysis of longitudinal studies found the odds ratio for an association between SEP and increased duration was larger than the odds ratio for association with increased incidences, arguing against a causal role for SEP [6]. However, the findings from a seven-year follow-up of the effect of changing SEP were consistent with a causal role for low SEP: one year increases in economic hardship (financial strain, deprivation and poverty) were associated with an increase in risk of MDD [7].

The relationship between SEP and MDD is complex. It is possible that low SEP may cause psychiatric illness, that those with psychiatric illness may cause sufferers to have lower SEP, or a form of social selection may act, preventing the attainment of a social position that could otherwise be expected on the basis of parental background [2], [8], [9], [10], [11]. Determining the relevant contribution of each factor requires longitudinal data, with multiple assessments preferably starting before the onset of illness. Few studies acquire suitable data, and even then the interpretation is complex [12].

Most of our understanding of the relationship between SEP and depression comes from studies in European populations. It is not clear that to what extent these findings can be extrapolated to other countries, either because of cultural differences (as has been suggested to explain the lack of an association between educational status and MDD in Japan [13]) or because of changing economic circumstances [14]. In a previous study from an early phase of our project, we examined the relationship between educational attainment and MDD in 4,000 Chinese women [15]. In this report we re-examine this relationship in a much larger and final sample and consider various interpretations of our results.

## Method

### Ethics Statement

The study protocol was approved centrally by the Ethical Review Board of Oxford University (Oxford Tropical Research Ethics Committee) and the ethics committees in all participating hospitals in China. The study was passed by all the ethics committees of the hospitals (attached a list of the names and addresses of hospitals is in supporting Information part). Written consent was obtained. All ethics committees approved this consent procedure. Major psychotic illness was an exclusion criterion, and the large majority of patients were in remission from illness (seen as out-patients). All interviewers were mental health professionals who are well able to judge decisional capacity. The study posed minimal risk (an interview and saliva sample).

### Study Subjects

Data for the present study draw upon the ongoing China, Oxford and VCU Experimental Research on Genetic Epidemiology (CONVERGE) study of MDD. Cases were recruited from 53 provincial mental health centers and psychiatric departments of general medical hospitals in 41 cities in 19 provinces and four central cities: Beijing, Shanghai, Tianjin and Chongqing. All cases were female and had four Han Chinese grandparents. Cases were excluded if they had a pre-existing history of bipolar disorder, any type of psychosis or mental retardation. Cases were aged between 30 and 60, had two or more episodes of MDD, with the first episode occurring between 14 and 50 and had not abused drug or alcohol before the first episode of MDD.

Controls were screened by personal interview to ensure that they had not experienced a prior depressive episode. To reduce the probability that controls might go on to develop MD, the minimum age for controls is 40. Controls were recruited from patients undergoing minor surgical procedures at general hospitals or from local community centers. Controls were chosen to match the region of origin of cases, were aged between 40 and 60, had never experienced an episode of MDD and were not blood relatives of cases.

All subjects were interviewed using a computerized assessment system, which lasted on average two hours for a case. All interviewers were trained by the CONVERGE team for a minimum of one week in the use of the interview. The interview includes assessment of psychopathology, demographic and personal characteristics, and psychosocial functioning. Interviews were tape-recorded and a proportion was listened to by the trained editors who provided feedback on the quality of the interviews.

### Measures

The diagnoses of depressive (dysthymia and major depressive disorder) and anxiety disorders (generalized anxiety disorder, panic disorder with or without agoraphobia) were established with the Composite International Diagnostic Interview (CIDI) (WHO lifetime version 2.1; Chinese version), which classifies diagnoses according to the Diagnostic and Statistical Manual of Mental Disorders (DSM-IV) criteria [16].

The interview was originally translated into Mandarin by a team of psychiatrists in Shanghai Mental Health Centre, with the translation reviewed and modified by members of the CONVERGE team. Phobias, divided into five subtypes (animal, situational, social and blood-injury, and agoraphobia), were diagnosed using an adaptation of the DSM-III criteria requiring one or more unreasonable fears, including fears of different animals, social phobia and agoraphobia, that objectively interfered with the respondent's life. The section on the assessment of phobias was translated by the CONVERGE team from the interview used in the Virginia Adult Twin Study of Psychiatric and Substance use Disorders (VATSPSUD) [17].

Education was treated as an ordinal variable divided into seven levels: no education or pre-school (scored 1) primary school or below (scored 2), junior middle school (scored 3), senior middle school or Technical and vocational school (in China, they both take the same time to be finished) (scored 4), adult/radio/television schooling, evening education or junior college (scored 5), bachelor degree (scored 6), and master degree or above (scored 7).

Occupation was treated as an ordinal variable divided into five levels: looking for work or unemployed (scored 1), permanently disabled, only temporarily laid off or sick leave (their limited capacity, but still intermittent work) (scored 2), retired from a paid job (they had worked) (scored 3), going to school, keeping house/staying at home (their daily life and learning is uninterrupted) (scored 4), working now for pay (scored 5).

Social class was treated as an ordinal variable divided into five levels: other (scored 1), semi-skilled and unskilled workers (scored 2), skilled manual employees (scored 3), administrative personnel, minor professionals, clerical and sales workers (scored 4), executives, business owners, and major professionals (scored 5).

All interviews were carried out using a computerized system developed in house in Oxford and called SysQ. Skip patterns were built into SysQ. Interviews were administered by trained interviewers and entered offline in real time onto SysQ, which was installed in laptops used by interviewers.

### Statistical Analysis

Statistical analyses were performed using the software package SPSS 17.0 (SPSS Inc., Chicago, IL). χ^2^ tests were also carried out in R, using the simulate.p.value option to confirm the value obtained from the χ^2^ distribution [18]. We performed logistic regression analyses to estimate the association between educational attainment, occupation, social class, comorbid disorders, and key symptoms of MDD. Odds ratios (OR) and 95% confidence intervals were used to quantify the strength of associations. The statistical significance for all tests was set at P<0.05.

## Results

The remarkable changes in China over the last 30 years have brought profound changes to the living standards of its people, a fact that has to be incorporated into studies such as ours that have a cross-sectional design. Therefore we began our analysis of the relationship between SEP and MDD by examining the relationship between educational, occupational and employment status with age, to establish at the outset any potential confounding influence on further analyses.

**Figure 1:**
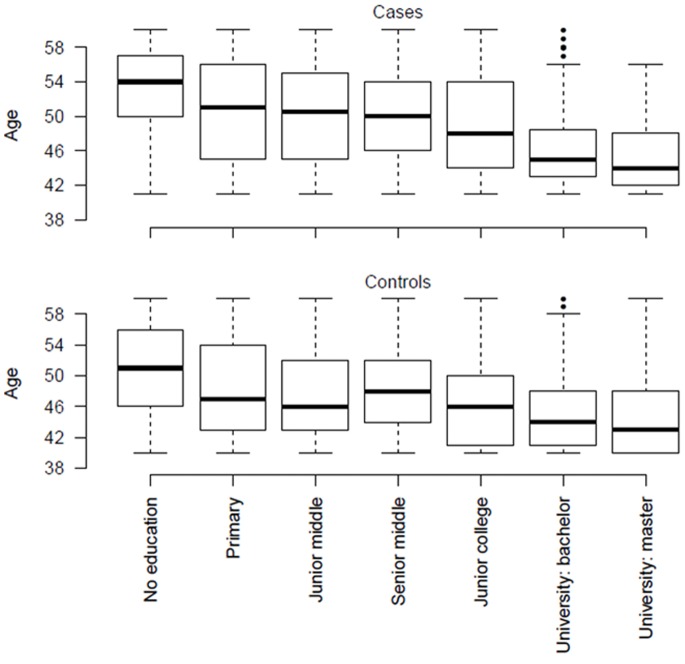
Relationship between age and educational attainment in cases of major depressive disorder and age matched controls.

As Figure 1 shows, there is a monotonic relationship between age and educational attainment: the younger age group is more likely to have attended University than the older group. The mean age of those who have been to university is 38, and the mean age of those who have only primary school education or less is 48. Since the age range for cases (30–60 years) was different from controls (40–60 years, imposed so as to reduce the inclusion of controls who might develop an episode of MDD) we censored cases so that the age range of cases and controls was the same. In all subsequent analyses we report results for age-censored data.

Table 1 shows the educational, occupational and employment status for 3,639 cases with MD and 3,800 controls. The distribution of two SEP measures differs significantly between cases and controls (education: χ^2^ = 77, df = 6, logP = 14.0; employment: χ^2^ = 111.9 df = 6, logP = 22.1). Social status shows a trend towards significance (χ^2^ = 8.4, df = 4, P-value = 0.07). We also obtained P-values by simulation for the χ^2^ tests. These results agreed with those from the χ^2^ distribution.

**Table 1:** Educational, social and employment status of the sample.

Educational attainment	Cases	Controls	OR		95% CI	P-value
No education or pre-school only	6.82	3.97	1			
Primary school	18.05	12.95	0.82		0.66–1.02	0.08
Junior middle school	31.27	32.92	0.55		0.44–0.68	3.49E-08
Senior middle school	29.24	30.26	0.56		0.46–0.69	1.01E-07
Junior college	8.85	11	0.62		0.49–0.78	6.79E-05
University: bachelor degree	4.92	7.89	0.62		0.49–0.80	1.80E-04
University: master degree or above	0.85	1	0.91		0.60–1.37	0.65
**Social status**	**Cases**	**Controls**	**OR**		**95% CI**	**P-value**
Semi-skilled and unskilled workers	9.21	11.31	1			
Skilled manual employees	28.52	26.2	1.22		1.05–1.41	6.12E-03
Administrative personnel, minor professionals, clerical and sales workers	17.24	14.1	1.15		0.98–1.36	0.08
Executives, business owners, major professionals	27.45	29.07	0.88		0.76–1.02	0.08
Other	17.57	19.31	0.87		0.74–1.01	0.07
**Employment status**	**Cases**	**Controls**	**OR**		**95% CI**	**P-value**
Working	22.81	50.45	1			
Temporarily unemployed	7.58	3.5	4.77		3.90–5.82	2.53E-52
Unemployed	1.1	0.79	2.85		1.86–4.37	1.80E-06
Retired	31.55	18.1	5.00		4.45–5.75	2.83E-111
Disabled	0.05	0.03	1.58		0.32–3.97	0.30
Keeping house/staying at home	30.97	19.31	3.44		3.06–3.86	3.02E-96
Other	5.94	7.79	1.47		1.24–1.74	1.24E-05
**Educational attainment**	**Cases**	**Controls**	**OR**		**95% CI**	**P-value**
No education or pre-school only	6.82	3.97	1			
Primary school	18.05	12.95	0.82	0.66–1.02		0.08
Junior middle school	31.27	32.92	0.55	0.44–0.68		3.49E-08
Senior middle school	29.24	30.26	0.56	0.46–0.69		1.01E-07
Junior college	8.85	11	0.62	0.49–0.78		6.79E-05
University: bachelor degree	4.92	7.89	0.62	0.49–0.80		1.80E-04
University: master degree or above	0.85	1	0.91	0.60–1.37		0.65
**Social status**	**Cases**	**Controls**	**OR**	**95% CI**		**P-value**
Semi-skilled and unskilled workers	9.21	11.31	1			
Skilled manual employees	28.52	26.2	1.22	1.05–1.41		6.12E-03
Administrative personnel, minor professionals, clerical and sales workers	17.24	14.1	1.15	0.98–1.36		0.08
Executives, business owners, major professionals	27.45	29.07	0.88	0.76–1.02		0.08
Other	17.57	19.31	0.87	0.74–1.01		0.07
**Employment status**	**Cases**	**Controls**	**OR**	**95% CI**		**P-value**
Working	22.81	50.45	1			
Temporarily unemployed	7.58	3.5	4.77	3.90–5.82		2.53E-52
Unemployed	1.1	0.79	2.85	1.86–4.37		1.80E-06
Retired	31.55	18.1	5.00	4.45–5.75		2.83E-111
Disabled	0.05	0.03	1.58	0.32–3.97		0.30
Keeping house/staying at home	30.97	19.31	3.44	3.06–3.86		3.02E-96
Other	5.94	7.79	1.47	1.24–1.74		1.24E-05

All of the three measures of SEP were found to have an influence on the risk of MDD. Odd ratios are shown in table 1. Large effects are seen for employment and education. Compared to being employed, any form of unemployment increases the risk of MDD. By dividing the sample into those in full time employment and those not, and examining the association with MDD in the two groups, we found an overall odds ratio of 3.14 for being unemployed (logP = 139, 95% CI = 3.1–3.75). The largest effect we found was for being retired, with an odds ratio of 5. Increasing levels education is a protective factor against MD. Any degree of education is protective. We compared those who had higher than primary school education to those who had primary school or no education. For those with higher than primary school education the OR for MDD is 0.60 (logP = 5.8, 95% CI = 0.50–0.74). Effects of social class are less marked. Compared to unskilled and semi skilled work, skilled employment brings some increase in risk, while there is a slight a reduction (non-significant) in risk for those in managerial posts.

We explored the reasons for the effect of education on MDD, since education is expected to be the key SEP variable determining the type of job and income obtained in later life. As Table 1 shows, MD cases contain an excess of individuals with low educational attainment: 25% of cases have only primary school education or less compared to 17% of controls.

We asked first whether lower educational attainment increases the number of episodes of MDD, rather than being associated with episode length, since in longitudinal studies the former has been suggested to indicate a causal relationship. We found that length of the longest episode was not associated with educational status (P-value = 0.55) but that the group with primary school education or less had experienced significantly more episodes (mean 6.58) compared to those who completed more than primary education (mean 5.16, P-value = 0.009). Mean ages of the two groups did not significantly differ, and there was no significant difference in the age of disease onset (P-value = 0.64), which otherwise might explain the difference in the number of episodes.

We examined the association with clinical features and educational attainment and present the results in table 2 for co-morbid psychiatric disorders. Co-morbid anxiety is commoner in those with more education (excepting generalized anxiety disorder); cases who have had more than primary school education are significantly more likely to have suffered from any of the anxiety disorders listed in Table 2 than cases with primary school education only (t = 4.79, P-value = 1.804e-06). Consistent with this, we find higher scores for neuroticism, a personality measure associated with susceptibility to anxiety, in the higher educational attainment group (t = −2.58, P-value = 0.01). Conversely, MDD is more severe in the lower educational attainment group: the number of A criteria endorsed is significantly higher (mean 8.5, compared to mean of 8.2, t = −7.24, P-value = 6.267e-13), and the rates of melancholia are higher (Table 2).

**Table 2:** Comorbid disease associated with educational attainment.

Comorbid disorder	Odds ratio	95% CI	P-value
GAD	0.98	0.92–1.04	0.49
Agoraphobia	1.10	1.02–1.20	**0.02**
Animal phobia	1.06	0.99–1.13	0.06
Any anxiety disorder	1.07	1.02–1.13	**0.01**
Situational phobia	1.07	0.99–1.15	0.08
Blood phobia	1.08	1.00–1.16	**0.04**
Social phobia	1.09	0.99–1.19	0.06
Panic	1.10	0.99–1.22	0.08
Melancholia	0.88	0.81–0.95	**0.002**
Dysthymia	1.11	1.01–1.21	**0.03**

The table shows the odds ratio for the presence of comorbid disease in patients with MDD. Odds ratios were calculated from a comparison between greater than primary school level education and only primary school or no education. An odds ratio greater than one means there is an increased risk of a comorbid anxiety disorder in individuals who have had more than primary school education. GAD is generalized anxiety disorder. CI is confidence interval.

## Discussion

Our analysis of the relationship between MDD and socioeconomic position has established the following: first, all three measures of SEP are associated with MDD; higher social status, being in work and having more education are associated with lower rates of MDD, consistent with their acting as protective factors. Second, we find that the phenomenology of MDD varies according to the degree of educational attainment: those with only primary school education are likely to have more episodes and to have a clinically more severe disorder, while those with higher educational attainment are likely to manifest comorbid anxiety disorders.

We have defined SEP in a relatively simplistic way, as consisting of differences in employment status, social class (as assessed by occupation), and education. Both occupation and education are recognized to be key components of social status [2], [19] and are commonly used to assess the relation between SEP and psychiatric disorders [20]. However we do not include data on income [21] or material possessions [12], variables that have also been used to explore this relationship for MDD [20]. Different assessments of socioeconomic status may translate into distinct concepts and confound interpretations of the relationship between disease and SEP, [22], as was recently shown in an analysis of the socio-economic determinants of depression: material possessions and living conditions, rather than spending, were found to be responsible for an association between wealth and MDD [20]. So while we argue our findings are robust, the nature of the association we report and the variables that drive it, will require more detailed analysis.

Our findings contribute to the large literature on the relationship between low SEP and increased rates of MDD (see for example [2], [7], [12], [21], [23], [24], [25]. We extend findings from Western society to China, confirming that as others have reported [26], [27], [28], the SEP indicators behave much as they do in the West. For example, the odds ratio we report for the effect of being unemployed (2.79) is very similar to that reported in the US National Comorbidity Survey (OR 2.54, CI 1.51–4.28) [29].

The clinical information we have obtained allows us to make two novel observations. First, we see that there are significantly more episodes of MDD among those with lower educational attainment (rather than longer episodes of disease), consistent with the hypothesis that the lower SEP increases the likelihood of developing MDD [6]. The effect we observe is relatively modest, and may be attributable to recall bias: the patients with the lower educational attainment have more severe illness and may therefore report more negative events in their life (such as repeated episodes of illness). However the effect is consistent with one large, seven-year longitudinal study [25].

Second, we find that different clinical features of MDD are related to variation in SEP. Higher educational attainment not only protects against MDD but alters its presentation, to a more anxious phenotype: there are higher rates of comorbid anxiety and higher neuroticism scores. It should be noted that our observation here does not contradict the finding that low SEP is associated with higher rates of anxiety disorder [12]; we are here only observing co-morbid anxiety in those already suffering from MDD. Our observation is also consistent with the association with the mild LCA class, which again indicates that SEP is somehow impacting on the clinical presentation of disease.

This raises an interesting question. As seen in our data (Figure 1), more people than ever before are attending university, acquiring the higher educational attainment that we suggest may alter disease presentation. One possible interpretation of our findings is that, over time, the nature of MDD in China will alter. Alternatively, increased access to higher education may be altering help-seeking behaviour: a particular set of clinical features in MDD may be associated with specific patterns of help-seeking behavior. This issue will require additional, preferably longitudinal, studies to resolve, but is a reminder of potential clinical impact of changing socio-economic patterns. In this respect, the rapid economic change in China provides a unique opportunity to explore the effects of changing socio-economic status on psychiatric illness.

Our results should be interpreted with respect to a number of important limitations. First, our patients, and many of our controls, were obtained from hospitals. In developed countries only about half of those with mental illness receive treatment [30] and it is likely that the proportion is even lower in developing countries. Our data are therefore drawn from an atypical population and our findings may not apply to community acquired samples. Furthermore we have only sampled women so our results may not apply to men. We do not know what extent the clinical findings we describe here are typical of other, non-Chinese settings.

Second, controls were selected at a different age from cases. Our study was designed to reduce the prevalence of MDD in our controls as much as possible; by selecting older controls we reduce the probability that the controls would later develop major depression. The older group will have passed through more of the risk period for the development of major depression. While we have incorporated this confound into the analysis, and we believe have minimized its impact, there may remain some unanticipated consequences that we are unaware of.

Third, our cross-sectional design makes it impossible to disentangle causal pathways. We would like to be able to distinguish between causation, social drift and social selection, but the our study design forbids this. At the moment the most we can say is that we have established the presence of this relationship in China. Finally, although we attempted to obtain matched controls, it is impossible to exclude all potential sources of bias (for example it is possible that those with higher levels of education are more likely to agree to participate in our study as controls).
